# Os Subepicondylare Mediale in the Elbow: A Radiographic Case Report of a Sesamoid Bone

**DOI:** 10.7759/cureus.61474

**Published:** 2024-06-01

**Authors:** Hamza Khan, Sumaira Noureen, Ambreen Shahid, Sana Khan, Ghazanfar Khan

**Affiliations:** 1 Department of Radiology, Mardan Medical Complex, Mardan, PAK; 2 Department of Surgery, Mardan Medical Complex, Mardan, PAK

**Keywords:** case report, radiographic evaluation, accessory bone elbow, sesamoid bone, os subepicondylare mediale

## Abstract

Sesamoid and accessory bones are small, oval-shaped structures that develop within tendons that pass over bony prominences. Although rare, accessory bones in the elbow region hold clinical significance because they can cause diagnostic uncertainty. We present the case of a 47-year-old previously healthy female patient who presented with left elbow pain and was discovered to have a sesamoid bone distal to the medial epicondyle of the humerus. On examination, the patient's left elbow was tender, but her range of motion was in normal range. Plain radiographs identified a small, oval-shaped bony fragment; further radiographic investigations were conducted. Computed tomography and ultrasound were performed to rule out diagnostic uncertainties. A diagnosis of os subepicondylare mediale, a rare sesamoid bone, was established through a thorough investigation of the well-circumscribed structure in accordance with the most current literature.

## Introduction

Accessory ossicles and sesamoid bones are small-shaped bony fragments in the human skeleton, considered to be normal anatomical variants [1]. A sesamoid bone is a small bone encased within a tendon or muscle, usually close to the joint areas. The term 'sesamoid' stems from the Arabic word ‘sesamum', reflecting the small size of most sesamoid bones [2]. They are usually encountered in the hand and foot, where they are well-recognized, but instances in the elbow are infrequent. There is uncertainty on whether the bones identified in the elbow are intra-tendinous sesamoids or accessory ossicles [3]. Wood and Campbell identified seven types of accessory ossicles, according to which types A and B are intraarticular, whereas types C, D, and G arise from ossification center failure to unite with the rest of the humeral condyle or the proximal end of the ulna, type E is apophyses that failed to unite with the humerus, and types D and F are classified as sesamoid bones, respectively [3, 4]. The research conducted by Vojtěch Kunc et al. introduced modifications to Wood and Campbell’s classification of accessory ossicles. Their study introduced the term 'os subepicondylare mediale' for type V accessory bones, contributing to categorizing the anatomical variations around the elbow joint more precisely [3]. The study conducted by Vojtěch Kunc et al. showed a prevalence of accessory bones of the elbow at 0.77%, with the most frequent variant being os subepicondylare mediale at 0.46%, positioned distal to the medial epicondyle [3]. Several pathological conditions have been noted to mimic sesamoids and accessory bones, such as osteochondritis dissecans, aseptic necrosis, avulsion fractures, synovial chondromatosis, traumatic fragments, articulated olecranon spur, calcareous bursitis, and persistent epiphysis. In theory, sesamoid bones might experience fractures, and inflammation, or interfere with typical joint function [5, 6]. Since accessory ossicles and sesamoid bones can resemble pathological disorders in the skeleton, it is crucial to correctly identify them in clinical practice.

## Case presentation

This is the case of a 47-year-old previously healthy female with a body mass index (BMI) of 23.9 kg/m^2^ who fell and injured her left elbow. She presented to us with a complaint of pain in her left elbow with no associated numbness or tingling.

On physical examination, the patient's left elbow joint was tender, more on the medial aspect, and pain aggravated with mobility and lifting heavy objects; however, her range of motion was in the normal range.

**Figure 1:**
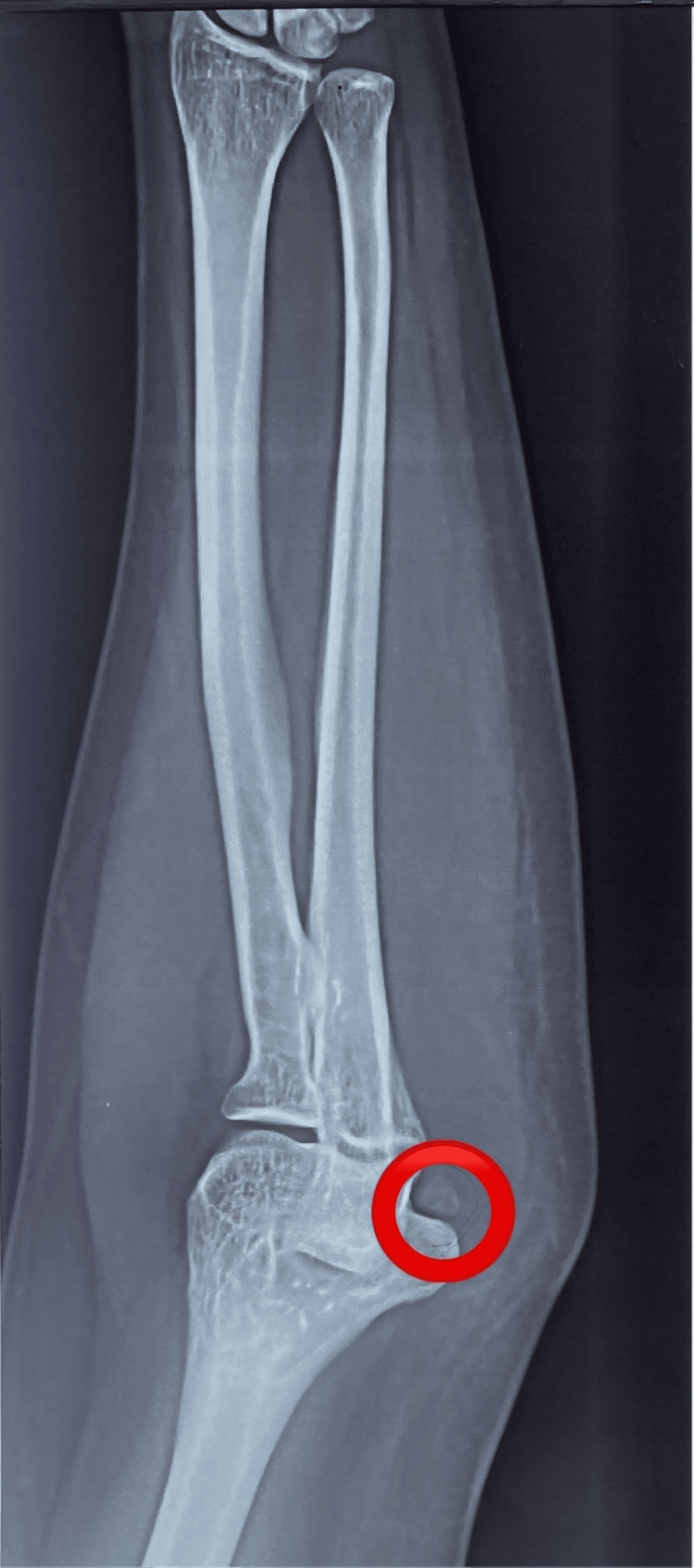
A plain X-ray (frontal projection) radiograph of the left elbow The red circle illustrates an accessory bone located beneath the medial epicondyle, featuring a distinctive oval shape and smooth border.

**Figure 2:**
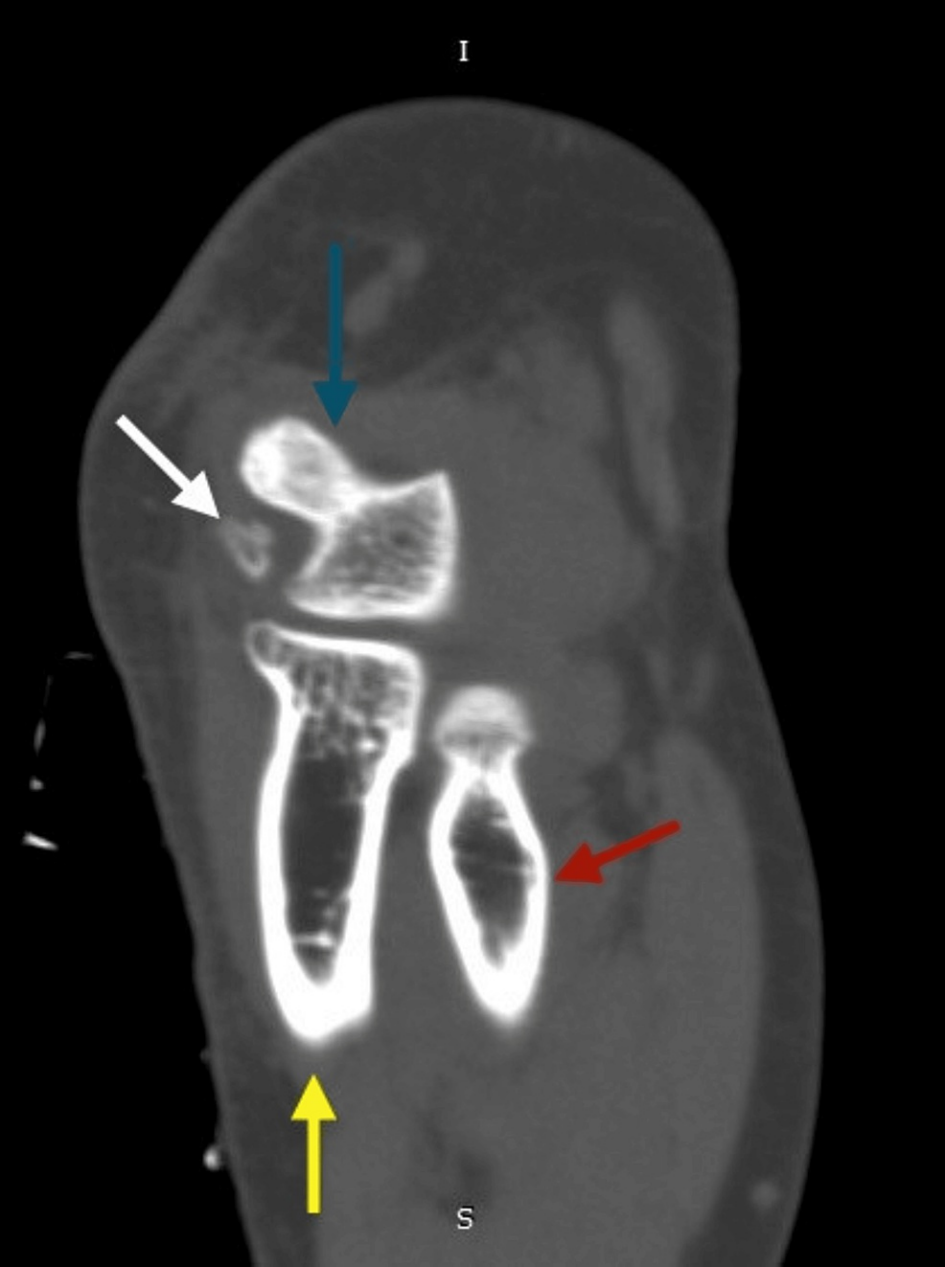
Computed tomography scan (sagittal view) of the left elbow joint The white arrow indicates the os subepicondylare mediale. The blue arrow indicates the medial humeral epicondyle. The red arrow indicates the radius. The yellow arrow indicates the ulna.

**Figure 3:**
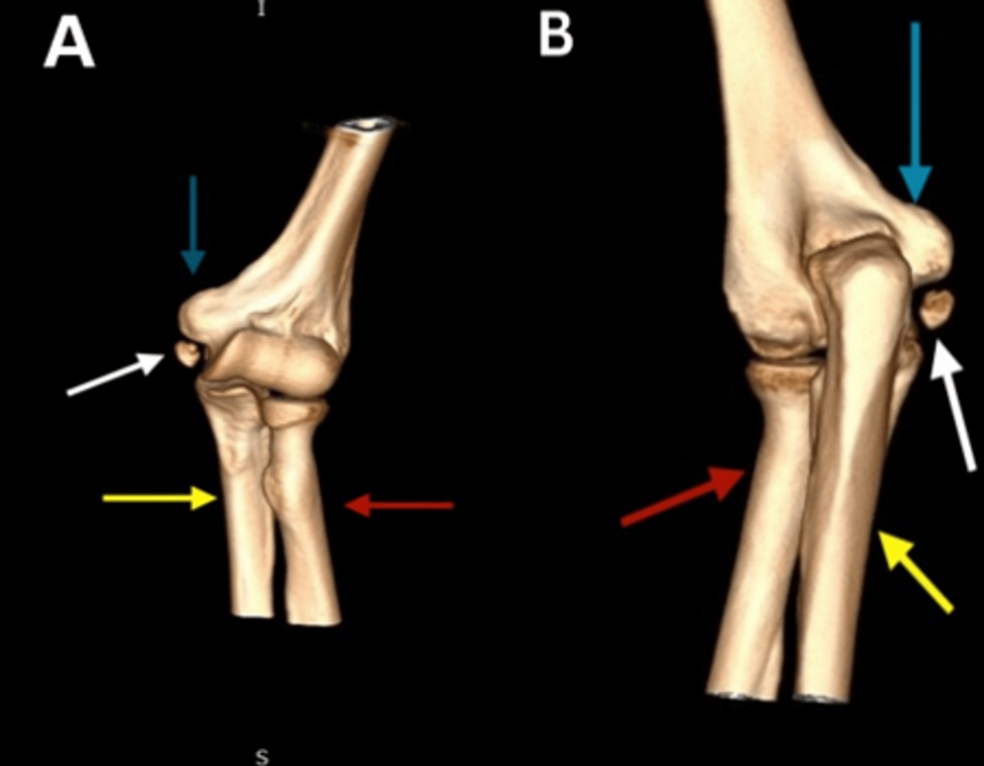
A 3D CT scan reconstructed a model of the left elbow joint (A) anterior view; (B) posterior view The white arrows indicate the os subepicondylare mediale. The blue arrows indicate the medial humeral epicondyle. The red arrows indicate the radius. The yellow arrows indicate the ulna.

**Figure 4:**
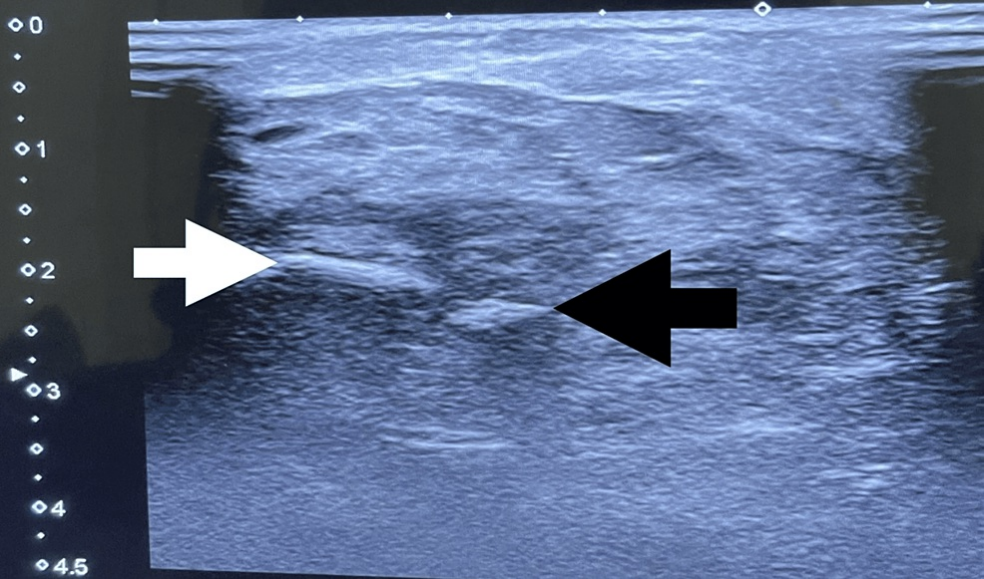
Ultrasonographic image of the left elbow (medial aspect) The black arrow indicates the os subepicondylare mediale and the white arrow indicates the medial epicondyle.

Beneath the left elbow's medial epicondyle, a bony fragment with an oval shape was identified on the plain X-ray radiographs (Figure 1). Computed tomography (CT) of the left elbow showed a small oval-shaped, well-corticated osseous structure distal to the medial epicondyle of the humerus (os subepicondylare mediale); no fracture or cortical irregularity was seen. Lateral and medial epicondyles, radial head, trochlea, olecranon process, and coronoid process were normal (Figure 2). Three-dimensional (3D) CT findings were consistent with a sesamoid bone (Figure 3). Ultrasonography revealed distinct connections between the medial epicondyle, trochlea, common flexor tendons, ulnar collateral ligament, and sesamoid bone (Figure 4). The patient's treatment plan consisted of the administration of oral non-steroidal anti-inflammatory drugs, if necessary for pain management, as well as the application of an elbow splint and exercise-ergonomics training.

## Discussion

Galen first coined the term 'sesamoids' to describe small, rounded, or oval digital bones that originate from their own ossification centers and are embedded entirely or partially in tendons or muscles [7]. Sesamoids mostly occur in the hands and feet, but they can also be found in other locations, among which are the pisiform bone in the wrist, the fabella in the knee, and the patella, the biggest sesamoid bone, but their incidence in the elbow is quite uncommon [5]. In a study conducted by Vojtěch Kunc et al., the sample showed a prevalence of accessory bones of the elbow at 0.77%, with the most frequent variant being os subepicondylare mediale at 0.46%, positioned distal to the medial epicondyle [3]. The sesamoid bones function like pulleys, assisting the strain on muscles and tendons and effectively reducing tension. This redistribution of forces not only ensures against injury but also strengthens the body's ability to bear weight and endure stress. These bones promote the body's biomechanics, enabling a wide range of motion [8]. If the sesamoids and accessory bones in the elbow joint are intra-articular, they may be susceptible to fragmentation after trauma, resulting in symptoms such as pain and restricted range of motion, leading to clinical and diagnostic challenges. Patients with sesamoids and accessory ossicles are subject to incorrect diagnoses, including osteochondritis dissecans, fractures, calcific tendinitis, and synovial chondromatosis [5].

In our case, a radiograph indicated an ovoid osseous structure adjacent to the trochlea with a subtle separating line. The distinct appearance prompted suspicions of either a fracture or osteochondritis dissecans. The CT scan confirmed an independent osseous structure (os subepicondylare mediale) situated between the medial epicondyle and the trochlea. The evident bone morphology affirmed the diagnosis of a sesamoid bone instead of a loose body. The dynamic ultrasound imaging ruled out ulnar nerve compression as the cause of pain, as the nerve displayed normal echogenicity and size. With the significant number of tendons present in the medial epicondyle region, it may be more precise to identify os subepicondylare mediale as a sesamoid bone, even though there is no clear interpretation established yet.

## Conclusions

The detection and description of the os subepicondylare mediale, a sesamoid in the elbow joint, as described in this unique radiographic case report, emphasizes the necessity of a thorough radiographic evaluation in detecting rare skeletal variants. This case report contributes to the limited existing literature on such anatomical variations in the upper extremity. Further studies and documentation on the prevalence, function, and clinical implications of this anatomical anomaly should be conducted to increase our understanding of elbow joint morphology and potentially improve diagnostic and treatment strategies for elbow-related pathologies.
